# Use of Preputial Skin as Cutaneous Graft after Nevus Excision

**DOI:** 10.1155/2010/951270

**Published:** 2010-10-11

**Authors:** A. D'Alessio, E. Piro, M. Brugnoni, L. Abati

**Affiliations:** Department of Pediatric Surgery, Azienda Ospedaliera, Legnano Hospital, 20025 Legnano (Milan), Italy

## Abstract

We report a four-year-old boy with a nevus covering all the plantar side of his second finger on the left foot. He was also affected by congenital phimosis. Surgical excision of the nevus was indicated, but the skin defect would have been too large to be directly closed. The foreskin was taken as a full-thickness skin graft to cover the cutaneous defect of the finger. The graft intake was favourable and provided a functional repair with good aesthetic characteristic.

## 1. Introduction

In order to prevent melanoma, selective removal of suspicious nevi is indicated. Furthermore, the site of lesion could indicate surgical excision to prevent continuous microtraumas [1–3]. Surgical excision could determine loss of substance due to the dimension of the nevus that could not be easily directly repaired.

The foreskin is a good autologous full-thickness skin graft in several conditions [4]. 

The authors report the use of foreskin as skin graft to repair a loss of substance due to excision of an interdigital nevus of the foot.

## 2. Case Presentation

**Figure 1:**
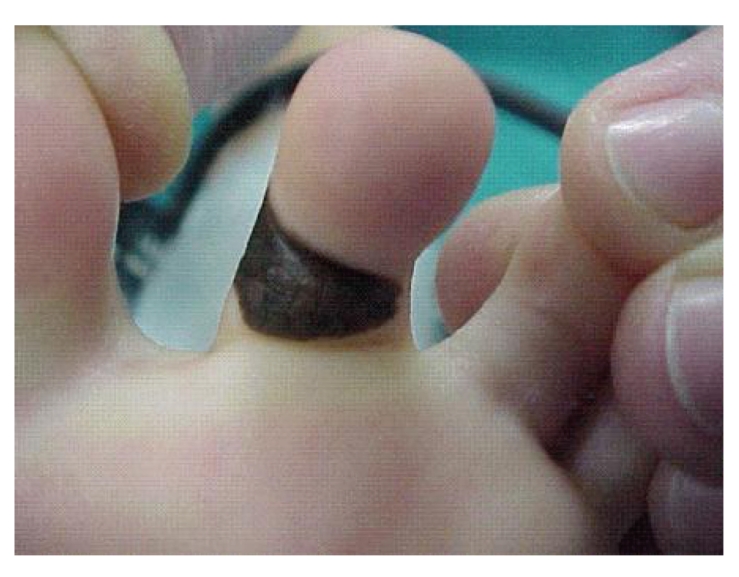
Site and features of the congenital compound nevus.

A four-year-old boy presented a 2 cm × 1.5 cm congenital compound nevus entirely covering the plantar surface of the second finger of his left foot (Figure 1). Paediatric dermatologist's indication was a radical excision because of the site and the dimension of this melanocytic lesion. Primary closure of the skin defect secondary to radical excision of the lesion was not indicated because of the large loss of substance and the risk of retractive scar. A skin graft was necessary to perform the repair. 

**Figure 2:**
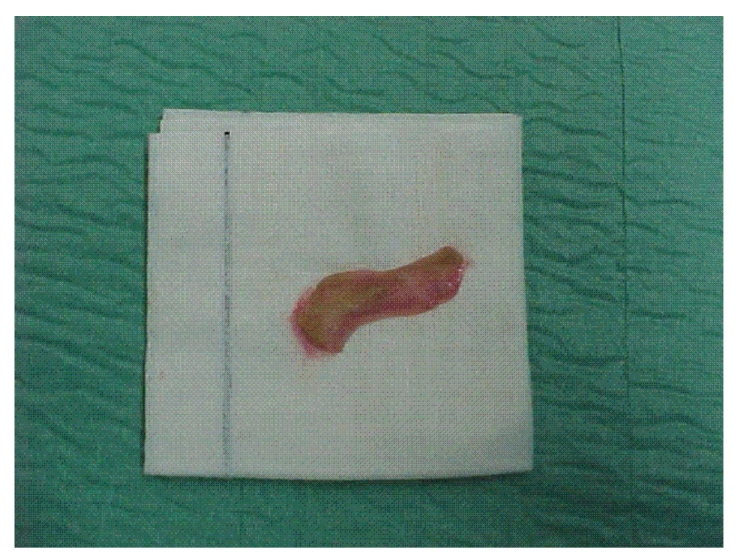
Foreskin trimmed in a rectangular shape.

**Figure 3:**
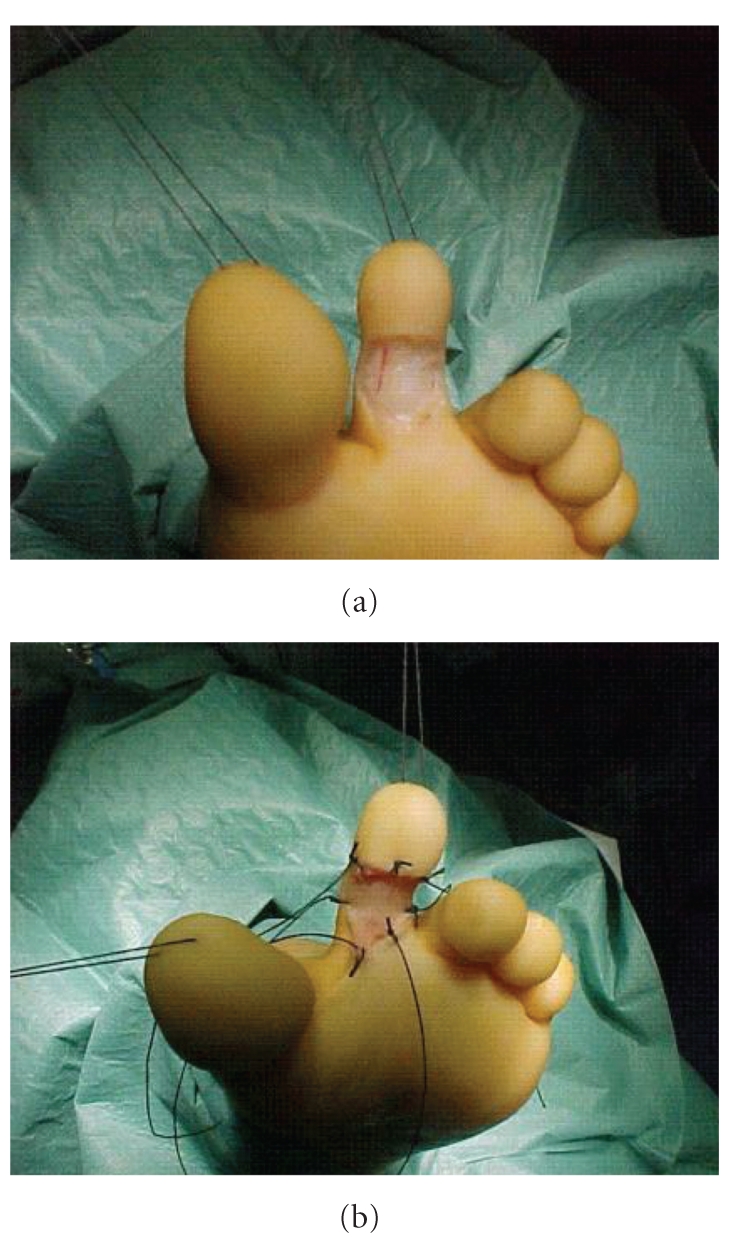
Residual open area after excision of the nevus (a) and foreskin graft sutured to cover the cutaneous defect (b).

The boy was also affected by congenital phimosis which required circumcision. So we decided to take foreskin as an autologous full-thickness skin graft. Then we performed circumcision and a radical excision of the nevus (Figure 3(a)); foreskin, trimmed in a rectangular shape (Figure 2), was sutured into the residual defect (Figure 3(b)). An occlusive medication was placed and removed ten days after.

**Figure 4:**
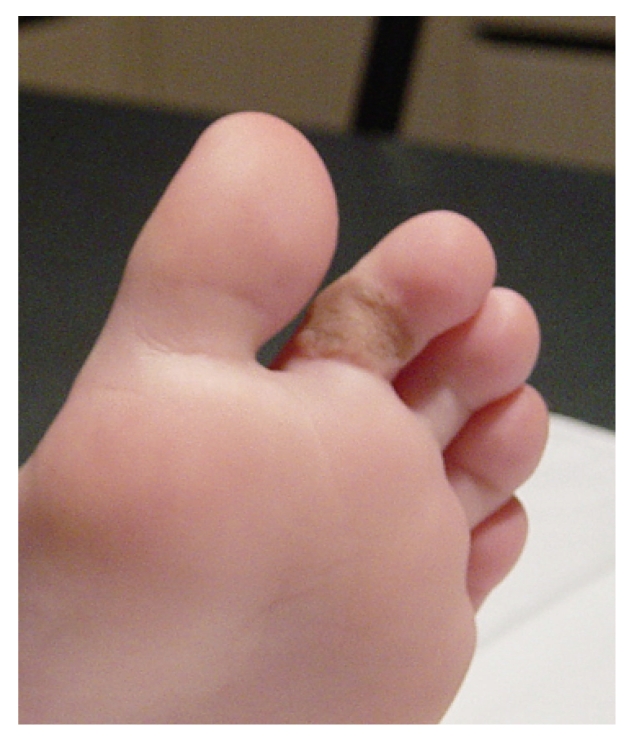
Delayed postoperative result (1 year after intervention).

Postoperatively the skin graft healed well. Today, one year after the operation, the patient has normal use of the foot finger with no evidence of contracture (Figure 4).

## 3. Discussion

Congenital melanocytic nevus is a frequent condition in childhood (0,2–1%) [1, 2]. The role of these lesions in increasing incidence of cutaneous melanoma is discussed and the prophylactic removal of all congenital melanocytic nevi is not supported: however, the most congenital melanocytic nevi are removed on preventing criteria. The selective excision of suspicious nevi is indicated when the features of a possible malignancy are faced. These features can include change in size or colour, irregular borders, or development of ulcerations. Other features that can justify excision are site and extension of the lesion, multinodular aspect, and the presence of other risk factors (immunodeficiency, dysplastic nevus syndrome, and xeroderma pigmentosa).

Excision of larger lesions require the use of local plasty, free tissue skin graft, or even the prior use of a tissue expander [3].Graft should be harvested from hairless areas where the skin is redundant (groin, volar wrist crease, volar elbow crease, and ulnar side of the hypothenar eminence). Foreskin as a source of skin graft [4] most often been used in urethral reconstruction for congenital or acquired penile defects [5, 6], in burn reconstruction [7], most commonly for eyelid resurfacing [8], and in syndactyly repair [9, 10]. 

Newborn circumcision remains controversial; this procedure has potential medical advantages (decreased risk of cancer of the penis and urinary tract infections) as well as disadvantages and risks (bleeding, infection, meatitis, and scarred phimosis) [11]. 

In Italy, neonatal circumcision is not routinely performed; this intervention is electively carried out until three years of age to repair congenital phimosis and at all ages in cases of scarred phimosis, recurrent balanoposthitis, and urinary infections. Therefore foreskin is frequently available as tissue graft in paediatric population. 

In our case, dimension and site (difficult to control) of melanocytic nevi justified the excision. Excisional biopsy determined a loss of substance that could not be directly restored. Foreskin was available because the boy was also affected by congenital phimosis, so we did not look for another source of skin graft. 

The most common problem reported after the use of prepuce as donor skin is hyperpigmentation. In our case, hyperpigmentation was not a contraindication for the use of foreskin as skin graft because the lesion was hidden localizated. 

Foreskin provides a skin of good elastic quality avoiding secondary retraction with a favourable rate of graft intake. Therefore, this source of graft gives the advantage of the absence of scar prejudice at the donor site.
